# The Effects of Caloric Restriction on Inflammatory Targets in the Prostates of Aged Rats

**DOI:** 10.3390/ijms25105236

**Published:** 2024-05-11

**Authors:** Vittoria Rago, Francesco Conforti, Daniele La Russa, Gemma Antonucci, Lidia Urlandini, Danilo Lofaro, Sabrina Bossio, Maurizio Mandalà, Daniela Pellegrino, Antonio Aversa, Silvia Di Agostino, Anna Perri

**Affiliations:** 1 Department of Pharmacy, Health and Nutritional Sciences, University of Calabria, 87036 Rende, Italy; vittoria.rago@unical.it (V.R.); lidiaurlandini@gmail.com (L.U.); 2 Pathology Unit, Annunziata Hospital, 87100 Cosenza, Italy; francesco.conforti@unical.it; 3 Department of Biology, Ecology and Earth Sciences, University of Calabria, 87036 Rende, Italy; daniele.larussa@unical.it (D.L.R.); gemma.antonucci@unical.it (G.A.); m.mandala@unical.it (M.M.); danielapellegrino@unical.it (D.P.); 4 de-Health Lab, Department of Mechanical, Energy, Management Engineering, University of Calabria, 87036 Rende, Italy; danilo.lofaro@unical.it; 5 Department of Experimental and Clinical Medicine, Magna Graecia University of Catanzaro, 88100 Catanzaro, Italy; sabrina.bossio@unicz.it (S.B.); aversa@unicz.it (A.A.); 6 Department of Health Sciences, Magna Græcia University of Catanzaro, 88100 Catanzaro, Italy; sdiagostino@unicz.it

**Keywords:** caloric restriction, aging, aged prostate, oxidative stress, inflammaging, fibrosis, EMT

## Abstract

Numerous animal models have demonstrated that caloric restriction (CR) is an excellent tool to delay aging and increase the quality of life, likely because it counteracts age-induced oxidative stress and inflammation. The aging process can affect the prostate in three ways: the onset of benign prostatic hyperplasia, prostatitis, and prostate cancer. In this study, we used 14 aged male Sprague Dawley rats, which were allocated into two groups, at the age of 18 months old. One group was fed ad libitum (a normal diet (ND)), and the other group followed a caloric restriction diet with a 60% decrease in intake. The rats were sacrificed at the age of 24 months. By immunohistochemical (IHC) and Western blot (WB) analyses, we studied the variations between the two groups in immune inflammation and fibrosis-related markers in aged prostate tissues. Morphological examinations showed lower levels of prostatic hyperplasia and fibrosis in the CR rats vs. the ND rats. The IHC results revealed that the prostates of the CR rats exhibited a lower immune proinflammatory infiltrate level and a reduced expression of the NLRP3 inflammasome pathway, together with significantly reduced expressions of mesenchymal markers and the profibrotic factor TGFβ1. Finally, by WB analysis, we observed a reduced expression of ERα, which is notoriously implicated in prostate stromal proliferation, and increased expressions of SOD1 and Hsp70, both exerting protective effects against oxidative stress. Overall, these data suggest that CR brings potential benefits to prostatic tissues as it reduces the physiological immune–inflammatory processes and the tissue remodeling caused by aging.

## 1. Introduction

Aging is a dynamic, physiological, and ineluctable process associated with a series of different changes at the molecular, cellular, and tissue levels, leading to a gradual decline in an organism’s capacity to maintain its homeostasis. The gradual loss of organ function and the reduced ability to regenerate increase the organism’s susceptibility and vulnerability to chronic diseases [1]. The hallmarks of aging include genomic hallmarks, strongly related to DNA damage, and non-genomic hallmarks, such as low-grade chronic inflammation, immune dysfunction, oxidative stress related to mitochondrial dysfunction, cellular senescence, and a reduced regenerative capacity [2,3]. Physiological aging is characterized by a condition termed inflammaging, consisting of a chronic state of increased sub-clinical levels of many proinflammatory mediators. A key contributor to inflammaging is the accumulation of senescent cells that exhibit a senescence-associated proinflammatory secretory phenotype [4]. Furthermore, aging is accompanied by progressive mitochondrial dysfunction which, in addition to promoting intracellular damage and an increase in ROS, causes the systemic spreading of mediators, termed mitokines, which strongly affects homeostasis [5]. A further crucial contributor to inflammaging is aged-related immune changes, termed immunosenescence, characterized by constitutively activated monocytes and T cells that, under basal conditions, secrete proinflammatory cytokines, such as TNFα, IL-1β, and IL6 [6,7]. The intricate process of inflammation is strictly linked to the activation of NLRP3 inflammasomes (multiprotein complexes belonging to innate immunity), which, triggered by pathogen- and danger-associated molecular patterns via TLR4, leads to the production of IL-1β, thereby initiating a potent inflammatory response [8]. Robust evidence describes NLRP inflammasome dysfunction during aging and suggests that the activation and hyperactivation of NLRP3 inflammasomes strongly contribute to inflammaging and the onset and progression of age-related diseases [9]. To strengthen this evidence, in vivo studies showed that NLRP3 pathway inhibition mitigates age-related organ damage [10,11,12].

Prostatic diseases such as hyperplasia and cancer are a consequence of glandular aging due to the loss of homeostasis [13]. The main pathological manifestations of benign prostate hyperplasia (BPH) are overgrowth and tissue remodeling. Prostate tissue remodeling is driven by lymphocytic and macrophage infiltration, leading to the secretion of many local proinflammatory cytokines, ROS production, and increased basic fibroblasts secreting TGF-β1. All these events determine stromal proliferation, transdifferentiation, and extracellular matrix production. The proinflammatory and profibrotic microenvironment causes a local vicious cycle, which can lead to the overproliferation of BPH nodules and concomitantly create a suitable microenvironment for cancer growth and progression [14,15,16].

The objective of anti-aging medicine, including both pharmaceutical and non-pharmaceutical approaches, is to decelerate the aging process and mitigate its associated effects. Caloric restriction, when implemented without malnutrition, has been demonstrated to have anti-aging effects and increase the lifespan in animal models. Furthermore, clinical studies have demonstrated that caloric restriction can be an effective therapeutic tool to reduce the incidence of age-related diseases and extend lifespan. Although the molecular mechanisms underlying the anti-aging effects of caloric restriction remain unclear, necessitating further research, it has been reported that energy restriction, in addition to having a positive impact on metabolism, has the potential to shape and modulate the immune response and, as a consequence, the inflammation and oxidative stress that represent the key drivers of pathologic prostate remodeling during aging [17,18].

Therefore, in this study, we chose an aged rat model to investigate the effects of caloric restriction on some non-genomic hallmarks of prostate aging, specifically focusing on inflammaging and fibrosis markers.

## 2. Results

### 2.1. Analysis of the Morphological Effects of Caloric Restriction on Aged Prostate Tissues

**Figure 1:**
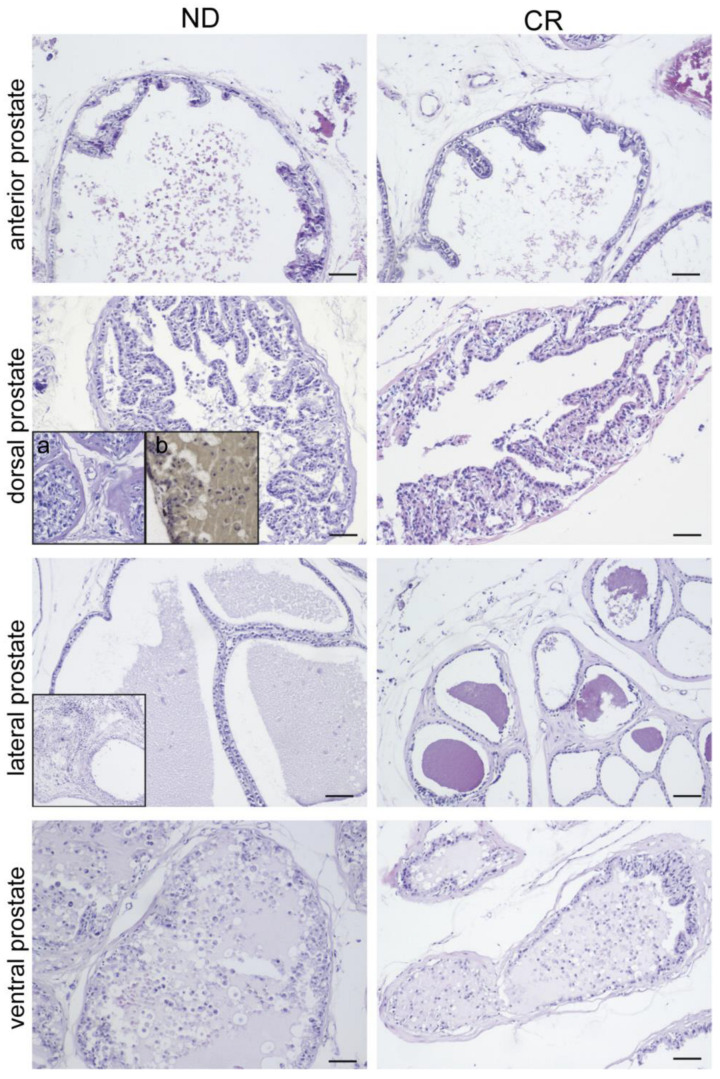
Histological features of prostate glands in ND vs. CR rats. Hematoxylin and eosin staining highlights an altered morphological appearance in the various prostatic areas in ND rats (**left panel**) compared with CR rats (**right panel**). The anterior prostate of the ND rat shows a large gland with a complex structure characterized by luminal projections (Ponte Romano) compared with the anterior prostate of the CR rat, where the glands have small basal cells arranged parallel to the basement membrane, sometimes with the architecture projected into the lumen. The dorsal prostate shows intraepithelial proliferation with moderate atypia (PIN) in the ND tissue, while in the CR tissue, the gland is hyperplastic without atypia. The ND inserts show (a) the presence of prostatic intraepithelial neoplasia confirmed by racemase positivity (b). In the lateral prostate, the ND image highlights a dilated gland with a complex architecture, focused on multiple layers with a moderate lymphomonocytic inflammatory infiltrate level (insert). In the CR rat, the gland presents small acini and fibromyomatous stromal hyperplasia. The ventral prostate in the ND rat shows a hyperplastic and focally atypical gland with dense secretion and a moderate lymphoplasmacytic inflammatory response. In contrast, in the CR rat, the gland presents focal hyperplastic aspects and secretion containing a mild lymphocytic infiltrate level. Scale bars: 50 µm.

We analyzed the impacts of caloric restriction (CR) on prostate tissues collected from 18-month-old rats subjected to a caloric restriction regimen for 4 months or fed ad libitum. In the prostatic tissues of control rats (ND) we detected, at a morphological level, the presence of some inflammatory foci associated with the presence of neutrophils, epithelial cells, and necrotic debris and many epithelial cells detached from the secreting epithelium and spilling into the prostatic glandular lumen (Figure 1; ND group). It is interesting to note that in many areas, the epithelium showed a greater number of epithelial layers than physiologically normal and alterations in the glandular architecture (Figure 1; ND group). In contrast, the prostate tissue morphological analysis in rats under caloric restriction (CR) showed an architecture of the gland very close to that of a physiologically normal gland and the morphology of the epithelium was almost normal, the infiltrates of inflammatory cells were at undetectable levels, and the subepithelial stroma was much more regular in its fibral arrangement (Figure 1—CR group; Table 1).

Notably, among the ND tissues, we detected prostatic intraepithelial neoplasia (PIN), a precancerous lesion strongly correlated with the in situ development of prostate carcinoma, characterized by the proliferation and dysplasia of the cells in the acini and prostatic ducts. The positive anti-racemase staining, a tumor marker detected in 64% of high-grade PIN [19], confirmed this observation (Figure 1; ND group).

### 2.2. Caloric Restriction Regimen Decreases the Presence of Inflammatory Cells in Aged Prostate Tissues

**Figure 2:**
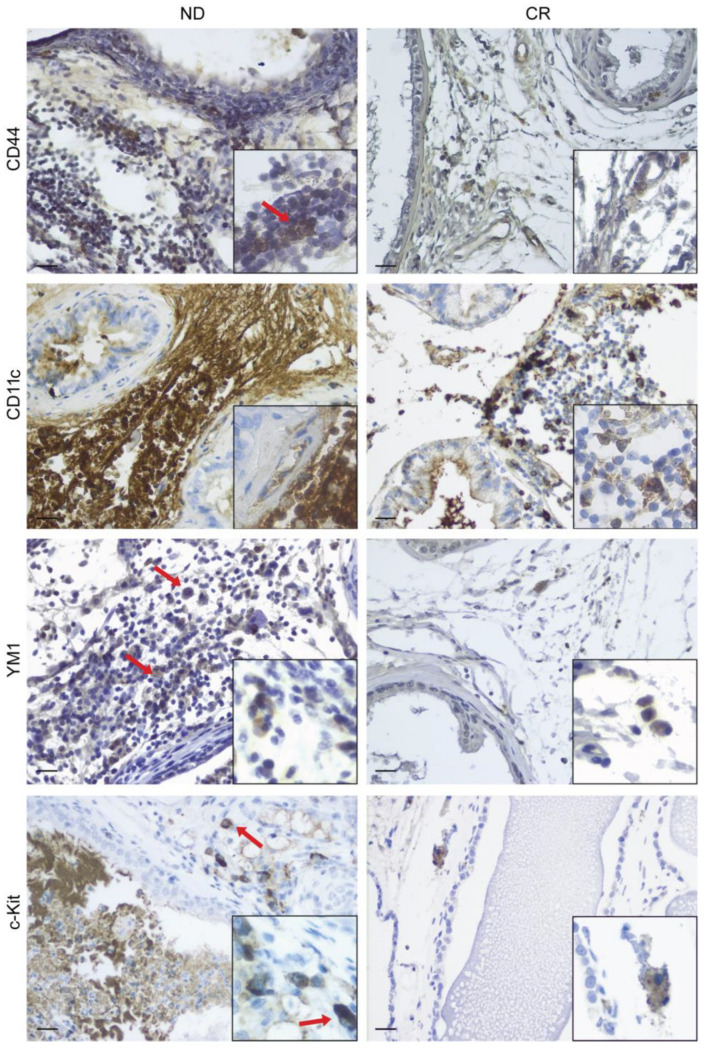
Immune cell infiltration in ND vs. CR prostates. The figure shows the different immunostaining expressions of a marker of T cell activation (CD44); proinflammatory M1 (CD11); anti-inflammatory M2 (YM1); and mast cells (c-Kit) in ND rat prostate (**left panel**) compared with CR rat prostate (**right panel**). The boxes in each image show a higher magnification. Red arrows indicate positive cells. Scale bars: 25 µm.

In this study, we performed immunostaining for the expression of cellular markers to determine a difference in immune cell infiltrates in the prostate tissues of rats subjected to CR or ad libitum feeding. In ND prostate tissues, we detected the significant positivity of the surface antigens CD44 and CD11c, which are cellular markers for activated T lymphocytes and monocytes/macrophages, respectively (Figure 2; ND group).

Conversely, in CR prostate tissues, the expression of these cellular markers in the stroma dramatically decreased (Figure 2—CR group; Table 1).

YM1 (chitinase-like protein 3 (Chil3)) is specifically expressed in rodents by M2-polarized macrophages and, in some conditions, by neutrophils [20]. The immunostaining revealed a higher expression of this cellular marker in the stroma of ND prostate tissues compared with CR ones (Figure 2; Table 1).

C-Kit is a receptor tyrosine kinase important for the differentiation and activation of mast cells [21]. Following the degranulation process, mast cells release proinflammatory mediators such as Il-1 and IL-6, and it is interesting to observe that the CR prostate tissues, in this case, showed a smaller number of cells compared with the control group (Figure 2; Table 1).

Collectively, these results suggest that CR induced a significant decrease in the immune cells’ infiltration into the glandular stroma of the prostate.

### 2.3. Caloric Restriction Regimen Modulates the NLRP3 Inflammasome Pathway in Aged Prostate Tissues

**Figure 3:**
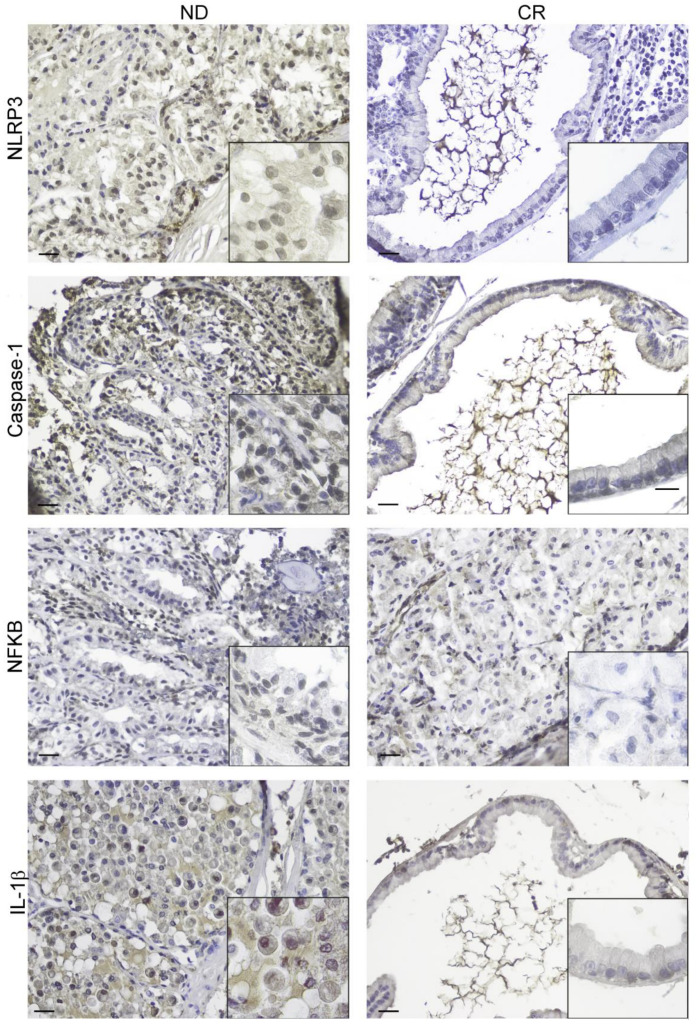
Immunohistochemical expression of proinflammatory markers (NLRP3, caspase-1, NFKB, and IL-1β) in rats on normal (**left panel**) and caloric restriction (**right panel**) diets. The boxes in each image show a higher magnification. Scale bars: 25 µm.

Considering the presence of many types of inflammatory cells, we analyzed the expression of some molecular components of the NLRP3 inflammasome pathway as it is also reported to be involved in the carcinogenesis of prostate tumors [22]. We analyzed NLRP3, caspase-1, NFKB, and IL-1β as they are members of the activation cascade of cellular events for NLRP3 inflammasome activation, as we reported in the enrichment pathway analysis for these four targets (Supplementary Figure S1). We documented that the expression of all four targets was downregulated in the CR prostate group compared with control tissues, strongly indicating that caloric restriction could reverse the inflammatory phenotype (Figure 3; Table 1).

### 2.4. Prostatic Fibrosis Shows Improvement under Caloric Restriction Conditions

Tissue inflammation is epidemiologically associated with the development of tissue fibrosis in many organs, including the prostate. We evaluated whether caloric restriction could have a role in the remodeling of the extracellular matrix (ECM) of the prostate tissues in our study.

**Figure 4:**
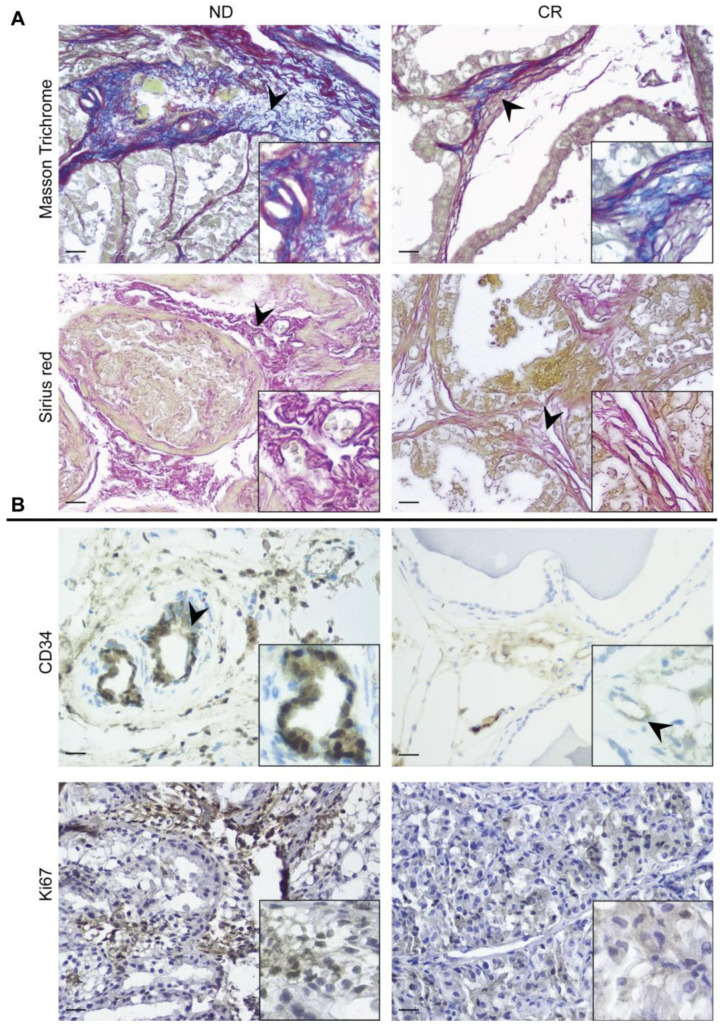
Prostate fibrosis: (**A**) Evaluation of the content of ECM (Masson’s trichrome) and collagen (Picrosirius red staining) in prostate tissues of ND and CR rats. (**B**) Immunolocalization of angiogenic (CD34) and proliferative markers (Ki67) in ND rat prostate (**left panel**) and CR rat prostate (**right panel**). The boxes in each image show a higher magnification. The black arrowheads indicate the increase in ECM and collagen in (**A**), while in (**B**), they show the different positivity of the endothelium. Scale bars: 25 µm.

We evaluated the phenotype of the ECM and the collagen content using Masson’s trichrome and Picrosirius red staining, respectively. These methods highlighted an extensive deposition of the ECM in the prostate tissues of the ND group, resulting in a dense fibrous tissue, which also determined the increase in stroma stiffness, validating the preliminary observation reported in Figure 1 (Figure 4). In the CR group, reduced levels of collagen deposition were detected compared with the control group, suggesting a decrease in the state of fibrosis (Figure 4).

CD34+ cells are important for endothelial regeneration but can also contribute to the inflammatory response and ECM remodeling and appear to be crucial for tissue fibrosis [23]. Accordingly, the immunostaining showed the presence of more perivascular CD34+ cells in the prostate tissues of rats fed ad libitum compared with those subjected to caloric restriction (Figure 4). Ki-67 is a marker of the proliferation of dysplastic and cancer tissues and for identifying the fibrotic phenotype [24]. Accordingly, Ki-67 expression showed a high number of positive cells in the stroma of ND rats compared with the prostate tissues from the CR group (Figure 4).

### 2.5. Caloric Restriction Attenuates Prostate Fibrosis by Inhibiting Epithelial–Mesenchymal Transition Players

**Figure 5:**
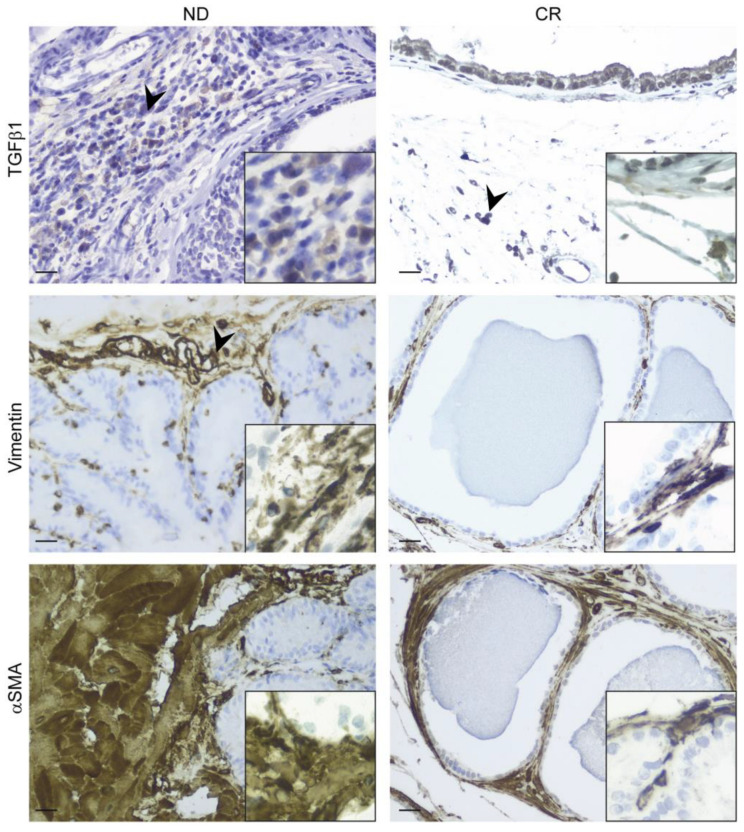
Immunohistochemical localization of stromal and myoepithelial alteration markers (TGFβ1, vimentin, and α-SMA) in rats on normal (**left panel**) and caloric restriction (**right panel**) diets. The boxes in each image show a higher magnification. Black arrowheads indicate positive cells. Scale bars: 25 µm.

The expressions of vimentin, α-SMA, and TGFβ1 have been reported as prognostic markers or therapeutic targets for different tumor types. Importantly, these three molecules are important players in the stiffening of the stroma during the epithelial–mesenchymal transition (ECM) of some tumor cells that begin the invasion of surrounding tissues and also in acute fibrotic processes [25]. We showed that the CR regimen decreased the expression of markers of stromal stiffening, particularly of α-SMA, compared with control prostate tissues (Figure 5; Table 1). These findings may suggest that CR was able to decrease the risk of matrix stiffening by turning off markers important for the EMT mechanism.

### 2.6. Caloric Restriction Enhances Prostate Antioxidant Defense Mechanisms and Mitigates Estrogenic Receptor Alpha Expression

**Figure 6:**
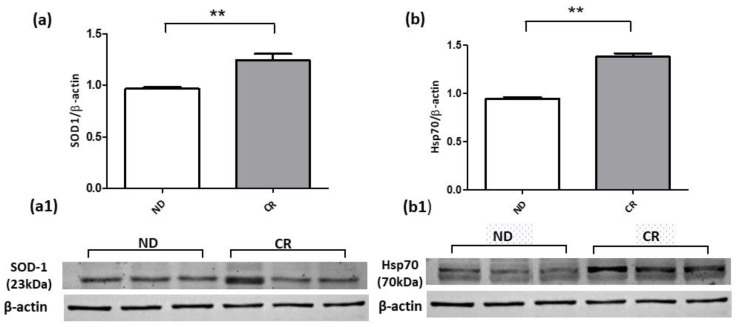
Representative Western blotting of SOD-1 (**a**) and Hsp70 (**b**) protein expressions in prostate tissue samples of ND and CR rats. (**a1**,**b1**) shows the blot band images. Protein loading was verified by using the anti-β-actin antibody. Data are means ± SE of three determinations for each animal (*n* = 3). Statistical differences were evaluated by *t*-test (** *p* < 0.01).

**Figure 7:**
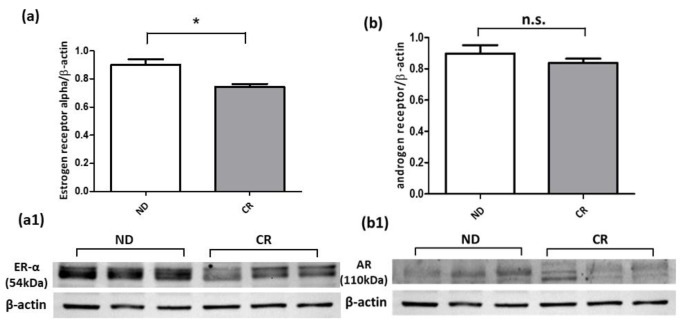
Representative Western blotting of ER-α (**a**) and AR (**b**) protein expressions in prostate tissue samples of ND and CR rats. (**a1**,**b1**) shows the blot band images. Protein loading was verified by using the anti-β-actin antibody. Data are means ± SE of three determinations for each animal (*n* = 3). Statistical differences were evaluated by *t*-test (* *p* < 0.05). n.s.: not significant.

To evaluate the effect of caloric restriction on cytoplasmic antioxidant defense mechanisms, we analyzed the protein expressions of SOD-1 and Hsp70, two important mediators in redox homeostasis. Our results clearly show that CR significantly upregulated the antioxidant enzyme SOD-1 in the CR group compared with the ND one (Figure 6a). A similar expression trend was also observed for the Hsp70 protein, whose expression level was enhanced by CR intervention (Figure 6b). The Western blot data of both the androgen receptor (AR) and estrogen receptor alpha (ER-α) proteins demonstrated that the AR protein was not affected by CR because its expression level was only slightly downregulated in CR animals compared with the ND experimental group (Figure 7a); on the contrary, the ER-α protein was significantly reduced in CR rats compared with the ND group (Figure 7b). Together, these results highlight the potential benefits of CR to the prostate’s redox/antiproliferative profile, which can contribute to mitigating the fibrotic process.

## 3. Discussion

Overall, the results collected in our study highlight that CR is able to reduce some of the pathological processes affecting prostate homeostasis during aging, such as inflammation and oxidative stress. Specifically, the prostates of old rats subjected to CR exhibited a lower level of hyperplasia and fibromatosis concomitantly with a reduced immune–inflammatory infiltrate and PIN foci number compared with ND rats.

Compelling evidence emerging from the literature suggests that a caloric restriction regimen accompanied by an adequate nutrient intake represents a promising therapeutic strategy to counteract the molecular processes driving aging and senescence, delaying the onset of several age-related chronic diseases and, consequently, extending lifespan [17,18]. The underlying mechanisms of CR are complex and multifactorial and impinge on many signaling pathways regulating growth, metabolism, the oxidative stress response, damage repair, and inflammation to modulate the aging process [26].

The prostate tissue microenvironment plays a role in producing autocrine and paracrine factors that guarantee organ homeostasis. Accumulating evidence strongly suggests that events related to stromal aging and senescence modify the prostate microenvironment, promoting cellular responses that contribute to the onset of organ pathologies, such as benign prostatic hyperplasia (BPH) and prostate carcinoma [13,15,27,28,29,30]. Over the years, several studies have focused on the physiopathological mechanisms involved in the onset and progression of age-related prostatic diseases. Several actors, including inflammatory mediators, hormones, dietary factors, and oxidative stress, are considered to have a key role in the development of BPH, but the primary actor is still unknown. There is almost unanimous consensus on the role played by non-genomic hallmarks of aging [2], such as oxidative stress and immunological inflammation, so it has been hypothesized that BPH is an immune-mediated inflammatory disease [31,32,33]. The origin of inflammation in the prostate is likely to be multifactorial, and it consists of a chronic process of wound healing sustained by the accumulation of immunocompetent cells secreting a high proportion of inflammatory cytokines, leading to the activation of hyperproliferative programs resulting in BPH nodules [14,34,35].

The first interesting finding that emerges from our study concerns the prostate tissue remodeling differences observed between the two groups of animals. In fact, the morphological analysis revealed that the prostates of rats subjected to CR exhibited a lower level of prostatic hyperplasia of glandular and stromal components compared with the ND group. Furthermore, a significant difference in both the quantity and the phenotype of the infiltrating immunocompetent cells was observed. Specifically, the immunohistochemical findings indicated that CR rats exhibited a significant decrease in T-lymphocyte infiltrate concomitant with a reduction in both proinflammatory (M1) and anti-inflammatory (M2) macrophages, and, interestingly, of infiltrated mast cells. Evidence emerging from preclinical and clinical studies suggests that infiltrated macrophages may have a role in BPH development and progression and that, in turn, BHP cells can also promote the occurrence of inflammation and favor its expansion by inducing more inflammatory cells to participate in inflammation, creating a local inflammatory microenvironment [33,36]. Interestingly, Qian and colleagues, reported that the subtype of polarized macrophage M2a could contribute to the development of BPH, possibly via modulating cell apoptosis, the cell cycle, the epithelial-to-mesenchymal transition (EMT), and fibrotic processes, as they secrete cytokines and growth factors, leading to the disease process of BHP [37].

Clinical studies have demonstrated the correlation between histological prostatic inflammation and prostate enlargement. The immunohistochemical study by Robert and colleagues, performed on surgically treated BPH specimens, showed that T-lymphocytes and macrophages were the main inflammatory cells infiltrating BPH tissues and highlighted that the prostate volume was significantly higher in patients with high-grade prostatic inflammation [38]. The critical role of inflammation in BPH progression was further confirmed by the MTPOS study, demonstrating that inflammation was more strongly associated with BPH progression than the American Urological Association Symptom Index and that inflammation has a greater role in men affected by conditions requiring anti-inflammatory medications [39]. Finally, the association of chronic inflammation with the severity and progression of BPH was subsequently confirmed by the robust and wide longitudinal REDUCE trial [40].

It is well known that mast cells can sense various “danger signals” via pattern recognition receptors, whose activation induces the release of a milieu of cytokines that alter the local tissue environment, leading to tissue fibrosis, repair, and remodeling [41,42]. Clinical studies have reported an increased number of mast cells in BPH tissues, and although it is not yet known whether they are functionally active, it has been suggested that mast cells could play an important role in the development and persistence of inflammation in BPH and in the correlated obstructive urinary symptoms [43]. To consolidate this finding, a recent in vivo study described that mice treated with mast cell inhibitors showed reduced prostatic fibrosis, less infiltration of immune cells, and decreased inflammation, emphasizing that within the immune–inflammatory milieu observed in BPH, mast cells may not only have a pathogenetic role but may also represent a potential therapeutic target [44].

A further interesting result that emerged from our immunohistochemical results is the evidence of a significant downregulation of the NLRP3 inflammasome belonging to the innate immune system and its downstream pathway, caspase-1, NFkB, and IL-1β in the prostates of CR rats compared with ND-rats. Different animal models of prostatic inflammation and BHP reported a significant upregulation of NLRP3, caspase, and downstream cytokines, IL-1β and IL-18, suggesting that inflammasomes may perpetuate the inflammatory state associated with BPH [45,46,47]. Furthermore, inflammasomes, by triggering inflammatory cascades, promote the formation of an inflammatory microenvironment that is conducive to tumor cell growth [48,49]. In support of this, the molecular mechanism by which the overactivation of the NLRP3 inflammasome promotes the malignant progression of prostate cancer has been defined, proposing this inflammasome as a new prognostic biomarker and potential therapeutic target for this cancer [22]. This evidence suggests that the reduced expression of the NLRP3 inflammasome pathway, together with the decreased overall immune–inflammatory milieu observed in CR rats, may also account for the lower incidence of foci of prostatic intraepithelial neoplasia observed in this animal model compared with the ND rat group. However, further molecular investigations are needed to sustain this hypothesis.

The Masson’s trichrome and Picrosirius red staining performed in our prostate tissue samples revealed a lower fibrotic component in the prostates of CR rats compared with ND rats. Fibrosis is an important factor in the onset and progression of BPH [50], and although the pathogenesis of fibrosis is not well understood, EMT is a well-known process that by facilitating the transformation of epithelial cells into myofibroblasts, instigates fibrosis in several organs, mainly during aging [51]. Recent studies have speculated that prostate enlargement may result from the accumulation of cells as a result of epithelial proliferation and the EMT process, as suggested by the evidence of a lower expression of the epithelial marker E-cadherin and a higher expression of the mesenchymal marker vimentin in BPH tissues, providing novel insight into the origin of stromal cells [52,53,54]. Our data suggest that the reduced fibrotic component observed in the prostate of CR rats may result from the CR-mitigated EMT, as we found a significantly reduced expression of the mesenchymal markers vimentin and αSMA in the prostates of CR rats compared with ND rats. Concomitantly, we observed a significant reduction in TGFβ1 staining, a profibrotic product of mesenchymal cells acting as a direct inducer of prostatic stromal hyperplasia and playing a role in several processes during BPH progression, such as the EMT and epithelial overproliferation [55,56,57]. Interestingly, the recent in vitro study by Shiyu Tong and colleagues showed that the modulation of TGFβ1 signaling efficaciously prevents TGFβ1-induced prostatic epithelial proliferation and the EMT, strongly suggesting that the profibrotic factor could have a crucial role in BHP’s etiology [58]. Furthermore, together with TGFβ1, we also detected a significantly reduced expression of c-Kit. It is well-known that the C-kit/SCF pathway is implicated in tissue remodeling and fibrosis [59]. As reported by Rojas, there is an aberrant molecular network where TGF-β signaling transcriptionally regulates the expression of the C-kit receptor ligand (SCF), and then SCF induces the TGFβ1 ligand via STAT3, thus forming a positive feedback loop between TGFβ1 and SCF/C-kit signaling [60]. The downregulation of EMT markers and its main inducer, TGFβ1, observed in rats subjected to CR could be due to the previously described reduction in the inflammatory milieu of proinflammatory cytokines, and the activation of NFkB signaling can trigger the EMT via various pathways [61,62]. The protective effect of CR against age-related EMT processes has been previously described, where CR suppressed the age-associated increase in pre-EMT gene, and prevented the local fibroblast formation in the senescent thymus, guaranteeing the maintenance of the thymic stromal cell microenvironment [63].

Among the complex physiopathological mechanisms underlying BHP, a role has been ascribed to AR and ER signaling as well as the ER/AR ratio. Clinical studies suggest that although serum testosterone levels decline during aging [64], androgen signaling may contribute to the occurrence and development of BPH [65,66,67]. Experimental data found that AR modulates the cellular growth of stromal cells by recruiting infiltrated macrophages [52,68,69,70] and that prostate epithelial AR function is important for the macrophage-mediated EMT and proliferation of prostate epithelial cells, highlighting that AR could have a role in the cross-talk between macrophages and prostate epithelial cells [71]. Similarly, epidemiological, animal, and in vitro studies strongly suggest a proliferative and proinflammatory role for ERα and an antiproliferative role for ERβ in BPH development [72,73]. The immunohistochemical analysis performed by Gangkak and colleagues on human tissues of BPH revealed a strong immunostaining for ERα in the stroma and for ERβ in the epithelium, ascribing ERα a role as a key mediator of estrogenic action in the prostate and BPH pathogenesis [72]. Starting from this evidence, we explored the protein expression levels of AR and ERα in protein extracts obtained from the prostate tissues of both animal groups. The immunoblotting analysis showed that CR, at least under the conditions and in the animal model we used, did not affect AR expression. Conversely, a significant reduction in ERα levels was observed in the prostate tissues of CR rats compared with the ND group, suggesting that CR could mitigate stromal proliferation by promoting the downregulation of stromal ERα signaling. However, to support this hypothesis, further investigations should be performed to study the ERα downstream pathway’s activation and verify whether CR reduces the expression of ERα mainly in the stromal compartment.

Inflammatory cells are the main source of reactive oxygen species (ROS) in the course of BHP. Oxidative stress, arising from the disruption of homeostasis between the production and elimination of oxidants, is another predisposing factor for BPH, as the DNA damage oxidative stress induces promotes compensatory cellular proliferation with the overgrowth of the prostate gland [74,75]. Furthermore, the impaired antioxidant system loses its ability to mitigate oxidative stress, exacerbating ROS-induced damage to prostatic tissues, demonstrating that BHP tissues exhibit a lower activity of superoxide dismutase (SOD) [76]. Robust data emerging from experimental aged models demonstrated that CR, in addition to improving the inflammatory response, efficaciously reduces oxidative stress by restoring the antioxidant defenses notoriously affected by aging [77,78].

The evaluation by Western blot analysis of the protein content of a key antioxidant enzyme, SOD1, revealed that CR enhanced the expression of SOD1. We can speculate that the latter finding may be a response signal by which CR protects prostate tissue against oxidative stress, as already reported in [79,80]. Recently, Scordino and colleagues described Hsp70/SOD1 signaling against oxidative stress damage [81]. The heat shock protein 70 participates in maintaining redox homeostasis through the activation of different intracellular signaling pathways, including increasing the expression and activity of SOD1 [82,83]. According to this evidence, our immunoblotting results revealed an increased protein expression of Hsp70 in CR prostate tissues, suggesting that CR may reduce oxidative stress by restoring SOD1 via Hsp70.

The prevention and treatment of age-related diseases are promising but challenging. Overall, medical combined with translational research is evaluating non-pharmacological therapies for the prevention and treatment of age-related diseases, such as CR, nutrition, exercise, and support of the intestinal microbiota.

Translating research findings into human medicine requires careful consideration of several factors, including the feasibility and safety of implementing calorie restriction in human populations, as well as its long-term effects on prostate health and overall well-being. Although some human studies have suggested the potential benefits of calorie restriction for prostate health, more research is needed to fully understand its effects and determine the optimal dietary interventions to promote prostate health and longevity.

One of the main limitations of our study was to confine the evaluation of the effects of CR on the prostates of old rats to tissue morphology and the protein expression of targets involved in inflammatory pathways. Certainly, the main future objective will be to carry out a broader evaluation of the aspect of transcriptional remodulation that CR could have in the in vivo model that we assessed and perhaps highlight other interesting pathways from the point of view of clinical targets.

## 4. Materials and Methods

### 4.1. Animals

The sample size was determined based on power analysis to ensure adequate statistical power to detect meaningful effects with a predefined level of significance. Specifically, we aimed to detect a difference of 40% with a power of >80% and a significance level of *p* > 0.05. This resulted in a calculated sample size of 14 animals. To minimize bias and ensure the validity of our results, a stratified randomization procedure was performed to ensure that each animal had an equal chance of being assigned to an experimental group. Therefore, the experiments were performed on 14 aged male Sprague Dawley rats (24 months old), which were housed in the animal care facility of the University of Calabria (Italy) in light- (12:12 h light–dark cycles) and temperature-controlled (22 °C) rooms and had free access to food and water. The animals at the age of 18 months old were divided into two subgroups: the normal diet (ND) group (n = 7) followed an ad libitum diet of a standard laboratory chow (Mucedola, diet 4RF21, Italy), and the caloric restriction (CR) group (n = 7) was fed a diet of the same chow restricted to 60% of the intake measured by weight in paired, control chow-fed rats. The calorie-restricted rats were fed every other day for a total period of 6 months, and the age of sacrifice was 24 months old. Food intake was recorded every other day, while body mass was recorded monthly. The animals were euthanized with inhalation of 4% isoflurane followed by cervical transection, and the organs studied were immediately removed and placed in cold HEPES–physiological saline solution (HEPES-PSS).

### 4.2. Prostate Tissues

Prostatic tissues were obtained after euthanasia, and each prostate was divided into two parts: a part of the tissue was immediately fixed in 4% neutral-buffered formalin and paraffin-embedded for histological and immunohistochemistry analysis; the other part of the tissue was rapidly rinsed with a 150 mM NaCl solution to remove excess blood and then stored at −80 °C until use.

### 4.3. Chemicals and Antibodies

The reagents were purchased from Sigma Aldrich (Milan, Italy) unless otherwise indicated. The following primary antibodies were used: anti-Ki-67, anti-NLRP3, anti-TGFβ1, anti-caspase-1, anti-NFKB, anti-IL-1β, anti-YM1, anti-SOD1, and anti-Hsp70 (Santa Cruz Biotechnology, CA, USA); anti-AMACAR, anti-αSMA, anti-vimentin, anti-CD34, anti-CD11, and anti-c-Kit (DAKO, Agilent Technologies, Milan, Italy); anti-phospho-CD44 (Cell Signaling Technology, Milan, Italy); and anti-AR and anti-ERα (Bio-Rad Laboratories, Inc., Hercules, CA, USA). Universal biotinylated horse IgG was used as a secondary antibody (Vector Laboratories, Burlingame, CA, USA). Masson’s trichrome kit and the Sirius red kit (Bio-Optica S.p.A., Milan, Italy) were used.

### 4.4. Histology and Immunohistochemistry Analysis

Morphological analysis and immunostaining of prostate tissue sections were performed on deparaffinized and hydrated serial sections, stained with hematoxylin and eosin, Masson’s trichrome, and Sirius red, using standardized protocols. Additional serial sections were used for immunohistochemical analysis and treated with the previously indicated antibodies. For each immunolocalization, the staining was optimized to ensure no background staining. Immunostaining was detected using diaminobenzidine tetrahydrochloride (DAB) followed by counterstaining with hematoxylin. Quantitative analysis of the stained sections was performed as previously described in [84]. For each staining, absorption controls were performed using primary antibodies pre-adsorbed with an excess of their purified blocking peptide at 4 °C for 48 h.

### 4.5. Imaging and Scoring

Prostate sections were analyzed using an Olympus BX41 microscope (Olympus Italia, Milan, Italy), and the images were taken with CSV1.14 software, using a CAM XC-30 for image acquisition. The scores for the immunoreaction were negative (0), weakly positive (1), moderately positive (2), positive (3), or strongly positive (4). The most frequent score among the three independent observers was chosen for each sample. For each slide, we evaluated a minimum of 100 cells and scored seven serial sections.

### 4.6. Western Blot and Densitometric Analysis

Prostate samples taken from each experimental group were rapidly lysed in ice-cold RIPA buffer (Sigma Aldrich, St. Louis, MI, USA) supplemented with a protease inhibitor cocktail (Sigma-Aldrich, Milan, Italy) and then centrifuged at 20,817× *g* for 20 min at 4 °C. The Bradford assay (Sigma, St. Louis, MO, USA) was used to evaluate protein concentrations, and the same amounts of total protein were separated via sodium dodecyl sulfate–polyacrylamide gel electrophoresis (SDS-PAGE gel) and then transferred to a nitrocellulose membrane (NitroBind, Maine Manufacturing, Kennebunk, ME, USA) using a mini transblot (BioRad Laboratories, Hercules, CA, USA). The membranes were then blocked for 1 h at RT with 5% non-fat dried milk in a 0.05% Tween-20 TRIS-buffered saline (TBS-T) solution and incubated overnight at 4 °C with the following primary antibodies directed against AR, ERα, SOD1, Hsp70, and β-actin (used as loading controls for protein normalization) followed by species-specific peroxidase-linked secondary antibodies (1:2000; Santa Cruz Biotechnology Inc., Dallas, TX, USA) for 1 h at RT. Immunodetection was performed with an enhanced chemiluminescence kit (Western Blotting Luminol Reagent, Santa Cruz Biotechnology Inc., Dallas, TX, USA), and the images were captured with the Invitrogen iBright FL1500 Imaging System. Digitalized immunoblots were subjected to densitometric analysis performed using ImageJ software (1.52a version, National Institutes of Health, Bethesda, Rockville, MD, USA).

### 4.7. Statistical Analysis

The Western blot data were analyzed by the Mann–Whitney test and unpaired Student’s test using GraphPad/Prism version 5.01 statistical software (SAS Institute, Abacus Concept Inc., Berkeley, CA, USA). Data are expressed as means ± standard error (SE). The intensity score was presented as the median (IQR) of the sample groups (ND and CR) and compared using the Wilcoxon test. A *p*-value of <0.05 was considered statistically significant. The analyses were conducted with R (4.3.1).

## 5. Conclusions

Overall, our data suggest that caloric restriction may represent a useful intervention in aging, as it could prevent age-associated diseases, such as prostate hyperplasia, and extend longevity. Our results support previous evidence demonstrating that CR has a pleiotropic effect modulating the main hallmarks of aging, such as immune inflammation and oxidative stress, which drive the tissue remodeling of the prostate while aging that notoriously increases the risk of prostate cancer development. However, further studies based on molecular approaches are needed to clarify the mechanisms underlying the protective effects of CR on aging-related diseases.

## Figures and Tables

**Table 1:** Immunoreactivity markers’ median scores (IQR) in rats subjected to normal diet and caloric restriction. * *p* < 0.05 vs. normal diet.

Marker	Normal Diet	Caloric Restriction
CD44	2.00 (1.25–2.00)	0.00 (0.00–0.75) *
CD11c	4.00 (3.25–4.00)	1.50 (1.00–2.00) *
CD34	3.00 (2.25–3.00)	0.50 (0.00–1.00) *
YM1	1.50 (1.00–2.00)	0.00 (0.00–0.75) *
c-Kit	3.00 (3.00–3.00)	0.00 (0.00–0.75) *
Ki-67	2.50 (2.00–3.00)	1.00 (0.25–1.00) *
NLRP3	3.00 (3.00–3.00)	0.50 (0.00–1.00) *
Caspase-1	3.00 (3.00–3.75)	1.00 (1.00–1.00) *
IL-1β	3.00 (3.00–3.00)	1.00 (0.25–1.00) *
NFKB	2.00 (2.00–2.75)	0.00 (0.00–0.75) *
α SMA	4.00 (4.00–4.00)	2.00 (2.00–2.00) *
Vimentin	2.00 (2.00–2.75)	1.00 (1.00–1.75) *
TGFβ1	1.50 (1.00–2.00)	0.00 (0.00–0.75) *

## Data Availability

The data can be shared upon reasonable request.

## Supplementary Materials

The following supporting information can be downloaded at https://www.mdpi.com/article/10.3390/ijms25105236/s1.
