# Supplementary material for: The Effects of Caloric Restriction on Inflammatory Targets in the Prostates of Aged Rats
Source: Int J Mol Sci. 2024 May 11;25(10):5236. doi: 10.3390/ijms25105236 (PMC11120753; doi:10.3390/ijms25105236)
Supplement: Supplementary file 1 [file ijms-25-05236-s001.zip › SFig1 legend.pdf]

Supplementary Figure S1: Pathway enrichment figure. Bar chart of top enriched pathways from the Reactome_2022 gene set library. The top 10 enriched terms for the input gene set are displayed based on the -log10 (p-value).
