# Supplementary figures and images for: The Effects of Caloric Restriction on Inflammatory Targets in the Prostates of Aged Rats
Source: Int J Mol Sci. 2024 May 11;25(10):5236. doi: 10.3390/ijms25105236 (PMC11120753; doi:10.3390/ijms25105236)

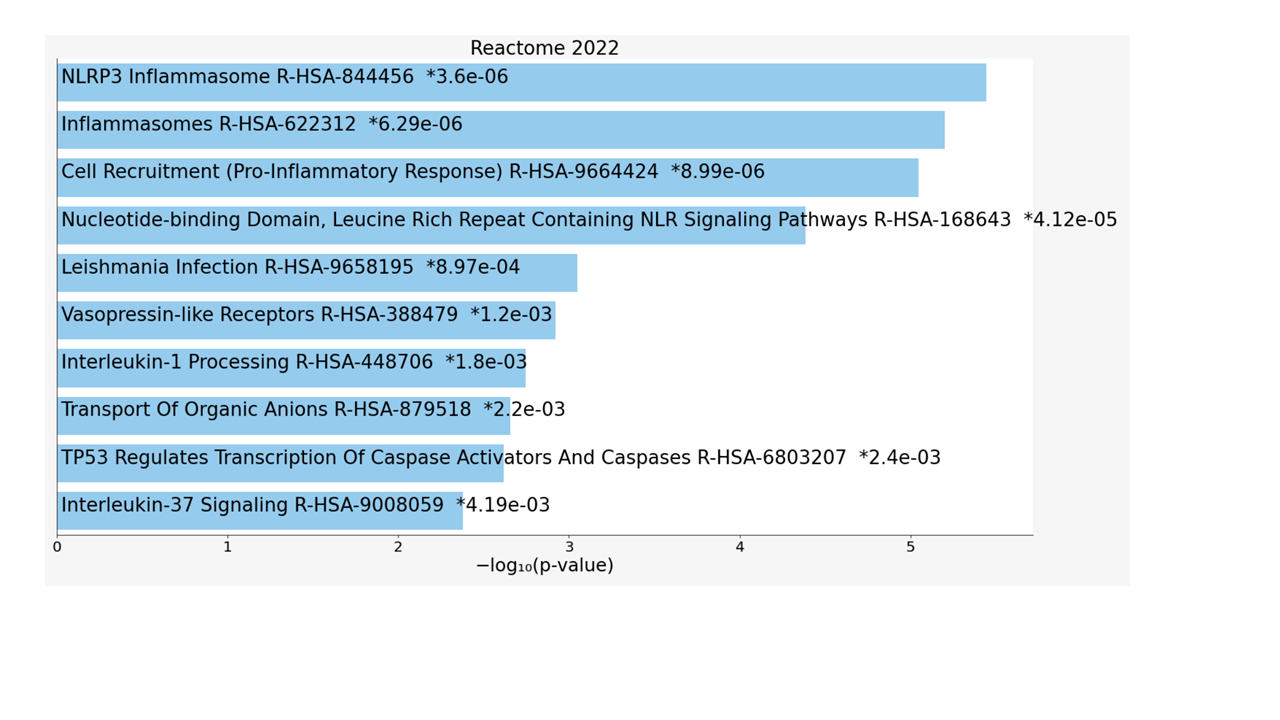

Supplement: Supplementary file 1 [file ijms-25-05236-s001.zip › SFig 1.tif]
